# Is There a Similarity in Serum Cytokine Profile between Patients with Periodontitis or 2019-Novel Coronavirus Infection?—A Scoping Review

**DOI:** 10.3390/biology12040550

**Published:** 2023-04-04

**Authors:** Archana Mootha

**Affiliations:** 1 Department of Biomaterials, Graduate School of Biomedical and Health Sciences, School of Dentistry, Hiroshima University, Hiroshima 739-0046, Japan; doctorarchanamds@gmail.com; 2 Department of Periodontics, Saveetha Dental College, Velappanchavadi, Chennai 600077, India

**Keywords:** cytokine, COVID-19, periodontal disease, immuno-pathology

## Abstract

**Simple Summary:**

This scoping review highlights the immune-related similarities between the COVID-19 infection and periodontal disease, especially focusing on serum cytokine levels, such as IL-1β, IL-6, and TNF-α levels. Overall, higher IL-1β, IL-6, and TNF- α levels were reported in COVID-19-infected patients compared to patients with periodontitis. However, most of the included studies indicated elevated serum pro-inflammatory cytokine (IL-1β, IL-6 and TNF-α) levels in diseases (COVID-19/periodontitis) compared to healthy controls included in the same study. This shows the strong immunopathogenic role of these proinflammatory cytokines in the rationale and progressive destruction of these two conditions, and this review aims to highlight the role of a robust immune response in inflammation progression and to educate the readers about the importance of oral hygiene during the pandemic era.

**Abstract:**

On 11 March 2020, the WHO declared a global emergency as a result of the ‘novel coronavirus infection’, which emerged from Wuhan, China, and rapidly spread across international borders. There is vast evidence that supports a direct link between oral cavities and this systemic circulation, but it is still unclear if oral conditions like periodontitis influenced the COVID-19 disease outcome. This scoping review highlights the fact that both periodontitis and COVID-19 independently increase serum pro-inflammatory cytokine levels, however there is a lack of documentation on if this biochemical profile synergizes with COVID-19 and/or periodontal severity in the same individuals. The purpose of this scoping review is to accumulate existing data on the serums IL-1β, IL-6, and TNF-α in COVID-19 and periodontitis patients and check if periodontitis negatively impacts the COVID-19 outcome, educating the population about the implications of COVID-19-related complications on their oral health, and vice versa, and motivating patients towards oral hygiene maintenance.

## 1. Introduction

**Figure 1:**
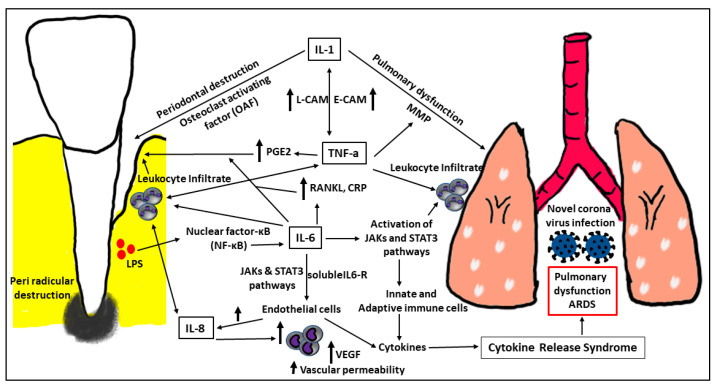
Cytokines released in pathogenesis of periodontal disease and the novel coronavirus infection. Figure legends: IL—Interleukin; L-CAM—Leukocyte cell adhesion molecules; E-CAM- Endothelial cell adhesion molecules; MMP—Matrix metalloproteinases; TNF—Tumor necrosis factor; PGE—Prostaglandin E; RANKL—Receptor activator of nuclear factor kappa-B Ligand; CRP—C-reactive protein; JAK—Janus Kinases; STAT—signal transducer and activator of transcription; VEGF—Vascular endothelial growth factor; LPS- Lipopolysaccharide.

Periodontitis is an inflammatory condition that damages the alveolar bone and soft tissues around the teeth, and compromises tooth stability [1]. It is one of the most common causes of partial to complete edentulousness; it remains a major contributing factor towards oro-functional abnormality and psychological challenges [2]. Periodontitis is established by the formation of a thin biofilm around the tooth necks, which induces inflammation caused by host-bacterial interactions and the production of pro-inflammatory cytokines. Following this, a strong immune response is initiated by the further secretion of inflammatory cytokines to combat pathogenic gram-negative bacteria, along with several adverse effects that manifest clinically (as tissue dissolution), immunologically (as increased immune cell infiltrate), and bio-chemically (as a pro-inflammatory cytokine milieu). Periodontal destruction is a sequelae of inflammation, vascular endothelial dilatation, leukocytic trans-endothelial migration, cytokine release, and chemotaxis, that cumulatively generates a continuous positive feedback loop (Figure 1). 

Increased vascular leakiness following periodontal microvascular dilatation causes cytokines to enter the systemic circulation, leading to systemic implications for periodontitis related to the heart, lungs, and brain apart from other complications such as pre-term birth, acute respiratory distress syndrome (ARDS), and “cytokine storm” as in the case of covid-19 infection. [3,4]. Periodontal inflammation is directed by an imbalance in the Th1/Th2/Th17 milieu [5]. A systemic link between periodontitis and bacterial pneumonia is established due to the accidental aspiration of periodontopathic bacteria into the respiratory tract, and some reports suggest that adequate oral hygiene maintenance could prevent the bacterial spread to the lungs [6,7]. Respiratory distress in COVID-19 leads to clinical hypoxia, which in turn increases reactive oxygen species that damage periodontal and lung tissues via apoptotic cell death [8]. Hypoxia also increases leucocytic infiltration and mast cell degranulation, which activates further chemokine secretion [9].

Cytokines that are frequently associated with periodontitis are IL-1β, IL-6, IL-12, IL-16, IL-17, IL-21, and TNF-α [10,11,12,13]. In particular, IL-1β, TNF-α, and IL-6 are secreted upon exposure to LPS from the P.gingivalis, which plays a pivotal role in bone resorption, peri-implantitis, and periodontitis apart from the role of these cytokines in other systemic inflammatory conditions, thus making these cytokines prime targets for therapeutic strategies [14,15,16,17], Along these lines, this scoping review aims to highlight if simultaneous increases in the serums IL-1β, IL-6, and TNF-α in periodontitis and COVID-19 infection patients could indicate an association between periodontitis and the coronavirus infection. 

### Research Questions

(i) If IL-1β, IL-6, AND TNF-α are elevated in the serum of patients with periodontal disease OR COVID-19 infection.

(ii) If elevated serum cytokines influence the severity of periodontitis or the COVID-19 infection.

## 2. Methodology

A detailed keyword search (Supplementary Table S1) in the PubMed, LitCovid, Cochrane, and Google Scholar databases was performed, and relevant articles were selected based on their titles and abstracts. Articles published on COVID-19 between December 2019 and March 2022 were included, whereas no time period was followed for the periodontitis articles. The full texts of the included studies were further scrutinized based on the inclusion criteria. Finally, the references of the selected articles were hand-searched to locate additional studies. 

Inclusion criteria.

1. Studies estimating IL-1β, IL-6, [AND] TNF-α in serum/plasma/blood/placental samples of patients affected with either the coronavirus infection [OR] periodontal disease.

Exclusion criteria

1. Studies estimating either of the serums IL-1β, [OR] IL-6, [OR] TNF-α.
2. Studies using patient samples for in-vitro analysis to estimate the release of IL-1β, IL-6 and TNF-α.
3. Samples where materials other than serum/blood/plasma/placental blood were used.
4. General reviews, systematic reviews, case reports, case series, and animal studies.
5. Analysis of the serums IL-1β, (or) IL-6, (or) TNF-α in patients with periimplantitis.
6. Articles published in languages other than English.
7. Studies which do not compare cytokine levels with control groups.

## 3. Results

**Figure 2:**
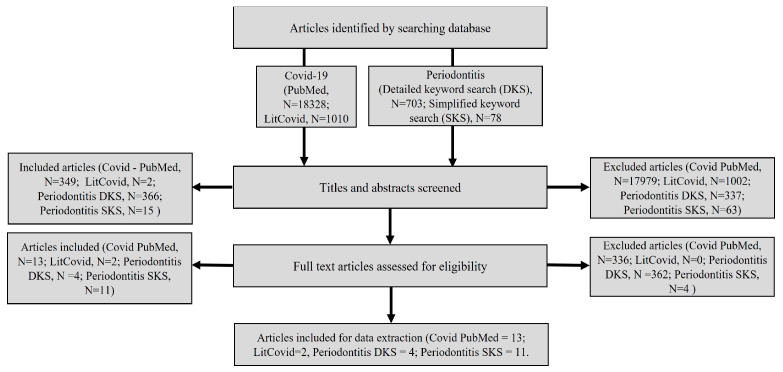
Flowchart of search strategy.

The search strategy for COVID-19 and periodontal disease yielded 19,338 and 781 articles, respectively, and were narrowed down to 351 studies and 381 studies on COVID-19 and periodontitis, respectively, based on their titles and abstracts (Figure 2). Systemic inflammatory conditions not only hasten disease severity but alter cytokine levels in periodontal and lung tissues; studies including patients with systemic conditions were excluded, and these excluded studies are shown in supplementary Table S2. Further full-text analysis based on the inclusion and exclusion criteria lead to the inclusion of 15 individual articles on COVID-19 and periodontitis. The included studies were critically screened to extract data, such as author name, country, journal, number of participants, study groups, study type, sample used for cytokine evaluation, and results of the serum/plasma cytokine (IL-1β, TNF-α and IL-6) levels (if reported). Since both Aggressive periodontitis (AgP) and Chronic periodontitis (CP) cases were included, studies with COVID-19 infection cases and controls of all of the age groups were also included, wherein three studies included participants aged < 18 y old [18,19,20].

**Figure 3:**
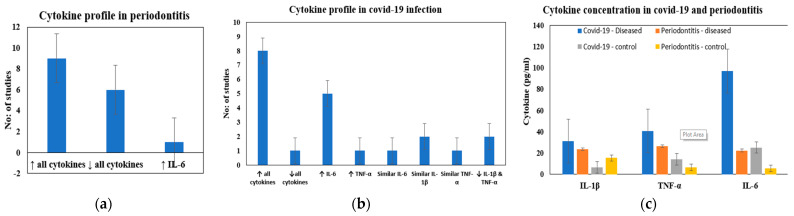
(**a**) Cytokine profile in periodontitis patients; (**b**) Cytokine profile in COVID-19 patients; (**c**) Comparison of cytokine levels between COVID-19 and periodontitis. (Arrows indicate increase or decrease in cytokine levels).

In the periodontitis group, nine studies showed all of the cytokines were elevated in periodontitis compared to the controls; six studies showed no difference between the controls and periodontitis, and one study only reported elevated IL-6 in severe periodontitis compared to the mild periodontitis group (Figure 3a) [21,22,23,24,25,26,27,28,29,30,31,32,33]. Data reporting methods were highly heterogeneous between the included studies; hence, further statistical analysis of these data was not possible. (Table 1)

Regarding COVID-19, eight studies showed increased IL-1β, IL-6, and TNF-α in these cases compared to the controls [18,19,34,35,36,37,38,39], five studies showed elevated IL-6 alone, and one study indicated higher TNF-α alone (Figure 3b) [40,41,42,43,44,45]. In addition, one study reported no significant difference between the cases and controls in all three of the cytokine levels, and one study reported no difference in all three IL-6, IL-1β, and TNF-α between cases and controls [41], and interestingly two studies showed decreased IL-1β and TNF-α in the cases compared to the controls [20,40,41,43,44,45]. A single study reported that in COVID-19 cases, high serum IL-1β and IL-6 in the disease were correlated with critical in-hospital deaths (*p* = 0.01) [36] (Table 2).

After the data analysis, serum cytokine concentration was extracted from 11 periodontitis studies and the highest reported concentration for IL-1 β level was 114 pg/mL, whilst IL-6’s was 125.4 pg/mL, and TNF-α’s was 202.71 pg/mL. Among COVID-19 patients, the highest reported serum concentration of IL-1 β level was 140 pg/mL, whilst IL-6’s was 249.0 pg/mL, and TNF-α’s was 151.59 pg/mL. Overall, higher serum IL-1 β, IL-6, and TNF-α levels were reported in COVID-19 compared to periodontitis, (Figure 3c). Another group of researchers suggested that patients showing IL-6 levels above the 50th percentile (IL-6 cut-off value above 163.4 pg/mL) had a 91.7% probability of dying (*p* = 0.0018), and TNF-α levels above the 50th percentile (cut-off level > 33.91 pg/mL) had a 75% probability of dying (*p* = 0.0648) [38]. On the other hand, a 1mm increase in PD is associated with a 25.06 pg/mL increase in IL-1β, a 1.72 pg/mL increase in IL-6, and a 1.70 pg/mL increase in TNF-α in saliva, supporting their role in tissue destruction [46,47]. Apart from these findings, it should be noted that IL-6 was the single cytokine that consistently increased in COVID-19 and was also used as a marker to distinguish levels of COVID-19 severity [41].

## 4. Discussion

Several periodontal studies indicated an increase in all three of the cytokines; however, a few studies found a decrease in the overall cytokine levels. Studies showing decreased cytokines were greater than the number of studies reporting increased IL-6 alone. Regarding COVID-19, most studies showed increased cytokines, whereas only one study reported a decrease in all cytokines. The number of periodontitis studies showing decreased cytokines was just over half of the number of COVID-19 studies showing decreased cytokines. Nevertheless, overall data supported the hypothesis that high IL-1β, IL-6, and TNF-α in the same patient play a role in COVID-19 and periodontitis pathogenicity independently. Interestingly, when studies reported only one cytokine increase, IL-6 increased more frequently than IL-1 and TNF-α. In fact, a few studies reported a decrease in IL-1β and/or TNF-α but not IL-6, which explains why IL-6 could be the sole determinant of severity in COVID-19 [20].

**Figure 4:**
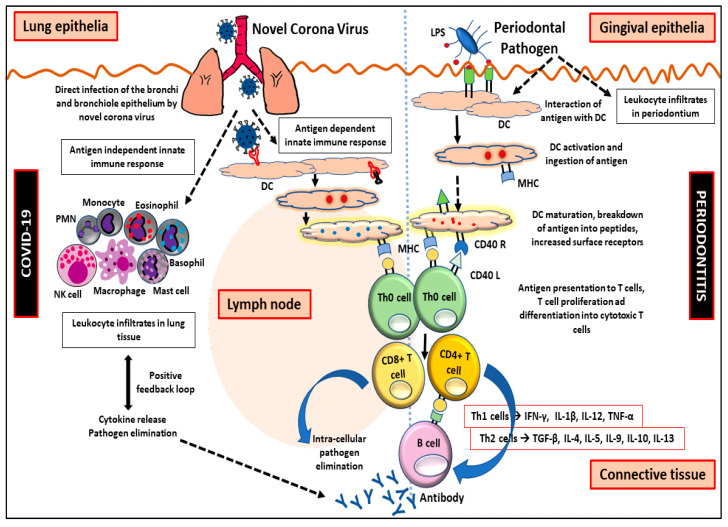
Immune-pathogenetic similarities and cytokine interplay in periodontitis and COVID-19.

Apart from periodontitis and COVID-19, local (salivary) and systemic (serum) TNF-α/IL-6/IL-1β levels also increased in other inflammatory conditions, such as chronic oral erosive lesions and ulcers, and these cytokine levels were inversely proportional to the IL-10 that regulated epithelial healing [48,49]. Interplay between IL-1β, IL-6, and THF-α allows for seamless disease progression. (Figure 4). For instance, TNF-α upregulates PMN infiltration, disrupts the epithelial integrity, and positively influences IL-1β and IL-6 to downregulate epithelial growth [50,51,52]. Following an epithelial breach, IL-1β and TNF-α increase gingival fibroblastic MMP and activate the protease pathway for tissue dissolution [53]. IL-6 and TNF-α interplay by raising IL-1 levels for advancing bone demineralization [54]. IL-1 β is largely associated with bone destruction, osteoclastic maturation, inhibition of ALP activity, and collagen synthesis [55,56]. In lung epithelium, bacterial LPS stimulate TNF-α to prime cells to produce more TNF-α and IL-1β to continue the destructive pathway [57].

Lastly, bacterio-viral interactions diminish respiratory ciliary activity and enhance bacterial adhesion, which facilitate pathogenic aspiration from oral cavities [58]. Table 3 shows that the occurrence of periodontitis is associated with COVID-19 and COVID-19 worsens the severity of periodontal disease and COVID-19-related hospitalization. Therefore, periodontal disease management and a decreased pathogenic load may decrease the accidental aspiration of periodontal pathogens into the lungs and decrease hospitalization or COVID-19-related complications [59].

## 5. Further Scope for Scrutiny

Currently, data reporting methods on the cytokine levels in periodontitis/COVID-19 are highly ambiguous, with limited available literature. The lack of homogeneity in the methodology, ethnicity, and sample size prevents generalizability and conclusions of a definitive outcome. Future studies with age-matched groups and control over possible confounding factors will lead to homogenous studies assessing the data with a more detailed meta-analysis. Table 4 enlists research areas that need focus to develop a further understanding of an association between periodontitis and COVID-19. 

Studies without control groups were not included in the methodology; hence, a large number of studies were excluded and the bias of the included studies has not been analysed, which are major limitations of this scoping review. In addition, the authors of the selected studies were not contacted to procure the serum cytokine values in the diseased patients when this was not mentioned in the manuscript, which might contribute to a skewed data analysis and could be an added limiting factor. 

## 6. Conclusions

Most studies showed greater increases in IL-6 in cases than controls, which suggests that IL-6 is a strong potential diagnostic and prognostic biomarker. However, this pattern has been more often reported in cases of COVID-19, and warrants additional research to conclude its role in periodontal pathology. Overall, serum pro-inflammatory cytokines are elevated in the presence of periodontopathic bacteria, so it could be derived that periodontal disease negatively influences the serum cytokine profile, which could further affect COVID-19 disease outcomes. Moreover, reports suggest that recent periodontal therapy/well-controlled periodontitis result in lesser COVID-19 complications. Furthermore, a two-way link is established between periodontitis and lung conditions due to the aspiration of oral bacteria into the respiratory tract and the dislodgement of coronavirus into the periodontal pockets. This may worsen the disease outcome of periodontitis and COVID-19 thereby underlining the importance of the maintenance of proper periodontal hygiene and prevent complications related to each other. 

## Figures and Tables

**Table 1:** Data collected from included studies from the periodontitis search strategy.

S No	Author, Year, Country	Journal	Study Type	No: of Participants, Age in Parentheses	Type of Periodontitis and Sample	Periodontal Assessment in Periodontitis Group	Cytokine Analysis	Results of Serum Cytokines	Periodontitis Group Cytokine Concentration (pg/mL)	Control Cytokine Concentration (pg/mL)
1	Fan Jiang, 2021, China	American Journal of Translational Research	observational study	CP = 86 (59.11 ± 8.63), HC= 60 (57.26 ± 7.15)	CP, Serum	BI, PD, PI, CAL, patients with more than 16 teeth	ELISA	All three cytokines were higher in CP than controls (*p* < 0.001)	NR in cases and controls	NR in cases and controls
2	R C Davies, 2011, UK	Journal of periodontal research	pilot study	AgP = 30 (36.7 ± 6.3) yrs), HC = 30 controls (18–45 yrs)	AgP, Serum	Radiographic bone loss (≥4–6 mm), CAL (≥2–8 mm), family history.	ELISA	No difference in all three cytokines between AgP and controls (*p* > 0.05)	IL-1β = 0.00 (0.00–0.02) IL-6 = 0.06 (0.00–0.19) TNF-α = 0.27 (0.00–0.72)	IL-1β = 0.00 (0.00–0.03) IL-6 = 0.06 (0.00–0.18) TNF-α = 0.24 (0.00–0.90)
3	T Fiorini, 2013, Brazil	Journal of periodontal research	randomized control trial	Case = 27 (18–35 yrs) Control = 30 (18–35 yrs)	Periodontitis, serum and GCF	PI, GI, supragingival calculus, cavities, overhanging restorations, BOP, PD, CAL.	Flow cytometry	No significant difference in cytokine levels between groups (all = *p* > 0.05)	median, IL-1β = 114.90 IL-6 = 5.40 TNF-α = 1.70	median, IL-1β = 134.15 IL-6 = 3.8 TNF-α = 1.15
4	Artem Eldzharov, 2021, Russia	Journal of Clinical Medicine	single-blind clinical trial	GCP = 40 (49.2 ± 4.3 yrs), Controls = 40 (49.7 ± 4.8 yrs)	moderate—severe chronic generalized periodontitis (Mild-moderate-severe), serum	PD ≥ 4 mm, and/or CAL ≥ 4 mm), generalized (>30% of sites), and radiographic vertical bone defect of at least 4 mm.	ELISA.	All three cytokines were significantly more elevated in GCP than controls (all *p* < 0.01)	IL-1β = 19.7 ± 3.2 IL-6 = 73.29 ± 5.11 TNF-α = 13.68 ± 2.39	IL-1β = 3.6 ± 1.01 IL-6 = 4.52 ± 0.81 TNF-α = 1.24 ± 0.22
5	Nidhi Medara, 2020, Australia	Cytokine	Investigational and interventional study	Periodontitis = 54 (53.28 ± 11.44 yrs), HC = 40 (49.30 ± 10.62 yrs)	Periodontitis type NR, serum and saliva	(PD) ≥ 5 mm, over 21 yrs of age, had a minimum of 16 teeth (excluding the third molars),	Luminex assay	Serum and salivary IL-1β, IL-6, and TNF-α were significantly higher at baseline in periodontitis than healthy and significantly decreased with treatment	NR in cases and controls	NR in cases and controls
6	Jennifer Chang, 2020, USA	Clinical Oral Investigations	Cross-sectional observational study	Periodontitis = 9 (52.2 ± 10.8 yrs), HC = 10 (52.8 ± 9.2 yrs)	generalized moderate to severe CP, Serum	Generalized moderate = >30% of teeth with BOP, CAL 3–4 mm, PD ≥ 5 and <7 mm, and radiographic bone loss of 16 to 30%; Severe = >30% of teeth with BOP, CAL ≥ 5 mm, PD ≥ 7 mm, and radiographic bone loss > 30%	ELISA	No statistically significant group differences in IL-1β, IL-6, and TNF-α between test and control groups	IL-1β = 4.42 (2.94–4.78), IL-6 = 3.99 (3.78–10.06), TNF-α = 11.82 (11.18–14.92)	IL-1β = 3.88 (2.69–5.25), IL-6 = 1.48 (0.82–2.98), TNF-α = 7.98 (4.16–14.62)
7	Helal F. Hetta, 2020, USA	Vaccines	Case-control study	Periodontitis = 55 (37.46 ± 4.2 yrs), HC = 20 (35.65 ± 1.6 yrs)	Stage two periodontitis, serum	PD ≥ 6 mm, (clinical attachment loss) CAL ≥ 5 mm, and radiographic evidence of bone loss in at least six teeth	ELISA	Significantly higher levels of serums IL-6, TNF-α, and IL-1β were seen in patients with CP than controls (*p* = 0.0001)	IL-1β = 84.02 ± 11.77, IL-6 = 125.4 ± 19.03, TNF-α = 202.71 ± 103.8	IL-1β = 7.03 ± 3.53, IL-6 = 19.03 ± 4.26, TNF-α = 11.01 ± 7.77
8	Xinling Wang, 2021, China	Oral Diseases	Case-control comparison study	Periodontitis = 36 (44.8 ± 11.3 yrs), HC = 25 (41.6 ± 9.7 yrs)	CP, serum	multiple sites with bone loss and PD > 4 mm	ELISA	Serum IL-1β, IL-6, and TNF-α were all remarkably more upregulated in CP group than control group	NR in cases and controls	NR in cases and controls
9	Dong-Hun Han 2020	Journal of periodontology	cross-sectional study	Healthy = 73 (40.85 ± 10.11 yrs); periodontitis = 20 (48.65 ± 9.27 yrs)	CP	community periodontal index (CPI): non-periodontitis(CPI 0 to CPI 2, including normal and gingivitis) versus periodontitis (CPI 3 or CPI 4)	ELISA	No difference in IL-1β, IL-6 and TNF-α levels between groups, but severe periodontitis showed higher IL-6 than control and mild periodontitis groups (*p* = 0.045)	IL-1β (ng/mL) = 0.35 ± 0.11, IL-6 (ng/mL) = 1.38 ± 0.21, TNF-α (ng/mL) = 2.10 ± 0.49	IL-1 β (ng/mL) = 0.47 ± 0.06, IL-6 (ng/mL) = 1.17 ± 0.12, TNF-α (ng/mL) = 1.92 ± 0.29
10	Tamires Szeremeske Miranda, 2019, Brazil	Clinical Oral Investigations	cross sectional study	Healthy controls = 25 (51.6 ± 7.2 yrs), CP = 26 (52.7 ± 8.3 yrs)	CP	>30% of sites with PD and CAL ≥ 4 mm and BoP, and a minimum of six teeth in each quadrant with at least one site with PD and CAL ≥ 5 mm and BoP	multiplex fluorescent bead-based immunoassay	No significant difference in cytokine levels between groups (all = *p* > 0.05)	IL-1 β = 0.9 (1.1 ± 0.8), IL-6 = 2.5 (2.9 ± 2.1), TNF-α = 3.5 (4.7 ± 2.9)	IL-1β = 1.1 (1.2 ± 0.6), IL-6 = 3.6 (3.5 ± 1.4), TNF-α = 4.9 (5.0 ± 1.7)
11	J. Bagavad Gita, 2018, India	Journal of periodontology	case-control study	Healthy controls = 66 (48 ± 4.413 yrs), CP = 66 (43.22 ± 1.951 yrs)	CP	Mild periodontitis = ≥2 sites with CAL ≥ 3 mm, PD ≥ 4 mm/one site with PD ≥ 5 mm; Moderate periodontitis = ≥2 sites with CAL ≥ 4 mm, or ≥2 sites with PD ≥ 5 mm; Severe periodontitis = ≥2 sites with CAL ≥ 6 mm and ≥1 site with PD ≥ 5 mm	ELISA	Serums IL-1β, IL-6, and TNF-α were all remarkably more upregulated in CP than control group (all *p* < 0.0001)	NR in cases and controls	NR in cases and controls
12	Mariana de Sousa Rabelo, 2021, USA	Cytokine	clinical study	Healthy controls = 15 (45.3 ± 7.9 yrs), CP = 15 (50.4 ± 8.1 yrs)	CP	Periodontitis: presence of ≥10 teeth with CAL ≥ 5 mm; ≥10 teeth with PD ≥ 5 mm; and ≥30% sites with BOP. HC = PPD ≤ 4 mm and BOP in <30% sites	multiplex fluorescent bead-based immunoassay system	No significant difference in cytokine levels between groups	IL-1β = 0.13 (0.03; IL-6 = 2.27 (0.95; 12.53), TNF-α = 1.35 (0.53; 4.84)	IL-1β = 0.09 (0.01; 0.25), IL-6 = 0.73 (0.44; 1.41), TNF-α = 0.33 (0.01; 2.73)
13	Hong Jiang, 2016, China	BMC Oral Health	Cross-sectional observational study	Healthy controls = 91 (26.53 ± 2.96 yrs), periodontitis = 442 (CP = 26.86 ± 3.63, severe CP = 26.61 ± 3.45 yrs)	CP	mild CP = PD > 3 mm or CAL > 3 mm; moderate CP = ≥4 with PD > 3 mm; severe CP ≥ 4 with PD ≥ 5 mm	ELISA	Serums IL-1β (*p* = 0.002), IL-6 (*p* = 0.052), and TNF-α (*p* = 0.005) were all remarkably more upregulated in CP than control group	IL-1β = 21.76 ± 2.51, IL-6 = 18.92 ± 1.97, TNF-α = 23.34 ± 2.56	IL-1β = 14.59 ± 3.13; 0.25), IL-6 = 16.12 ± 2.18, TNF-α = 15.18 ± 3.94
14	Ozlem Fentoglu, 2010, Turkey	Journal of Clinical Periodontology	Observational study	Healthy controls = 91 (31–54 yrs), periodontitis = 442 (31–54 yrs)	CP	Healthy: GI< 1, % BOP < 25%, No sites with CAL, CP = ≥4 teeth with a PD ≥ 5 mm, with CAL ≥ 2 mm	ELISA	Serums IL-1β, IL-6, and TNF-α were all remarkably more upregulated in CP than control group (all *p* = 0.006)	IL-1β = 2.94 (0.80–26.08), IL-6 = 5.82 (3.51–62.53), TNF-α = 14.82 (1.80–177.74)	IL-1β = 2.44 (0.71–27.07, IL-6 = 5.40 (3.20–22.70, TNF-α = 25.08 (0.71–1459.76)
15	Jin Zhang, 2017, China	American Journal of Orthodontics and Dentofacial Orthopedics	Comparative study	Healthy controls = 117 (33.9 ± 5.7 yrs), periodontitis = 52 (36.5 ± 6 5.8 yrs)	CP	pathologic tooth migration, tooth displacement, gingival bleeding, and mobility, PD, periodontal abscess, CAL = mild (1–2 mm, *n* = 48), moderate (3–4 mm, *n* = 39), and severe (≥5 mm, *n* = 30)	ELISA	Serums IL-1β, IL-6, and TNF-α were higher in periodontitis than healthy controls (all *p* < 0.05)	IL-1β = 11.69 ± 4.13, IL-6 = 7.92 ± 3.02, TNF-α = 17.68 ± 5.61	IL-1β = 1.47 ± 0.59, IL-6 = 7.92 ± 3.08, TNF-α = 4.97 ± 1.76

Table legends: CP = chronic periodontitis; AgP = Aggressive periodontitis; PD = pocket depth; CAL= clinical attachment loss; BI = bleeding index; PI = plaque index; GI = gingival index; BOP= bleeding on probing; GCP = generalized chronic periodontitis; GCF = gingival crevicular fluid.

**Table 2:** Data collected from included studies from the COVID-19 search strategy.

S No	Author, Year, Country	Journal	Study Type	No: of Participants	Results of Serum Cytokines	Cases Cytokine Concentration (pg/mL)	Controls Cytokine Concentration (pg/mL)
1	Tamara S. Rodrigues, 2021 Brazil	Journal of experimental medicine	Observational study	Covid + ve = 124 (59.25 + 18.01 yrs, mild, moderate, and severe), HC = 73	IL-6 increased more in cases than controls (*p* = 0.000), and there was an insignificantly greater increase in IL-1β in cases than controls; no significant increase in TNF-α in cases than controls (*p* = 0.3)	NR in cases versus controls	NR in cases versus controls
2	Lu Qingqing, 2021, China	International journal of clinical practice	Cross-sectional observational study	Covid + ve = 20 (8–78 yrs), HC = 35 (7–8 yrs),	IL-1β(*p* = 0.000), IL-6 (*p* = 0.000), and TNF-α (*p* = 0.000) of COVID-19 patients were significantly higher than control group	IL-1 β = 7.22(10.39), IL-6 = 7.56(10.89), TNF-α = 14.21 (23.80)	IL-1 β = 0.02 (0.00), IL-6 = 0.03 (0.00), TNF-α = 0.02 (0.00)
3	Shafiek Hala K, 2021, Egypt	Pediatric immunology	multi-center study	Covid + ve = 92 (10.5 (8.6–17.8 yrs) (Moderate = 68, severe = 18, critical = 6),HC = 100; (<18 yrs)	Cases had higher IL-1β, IL-6, and TNF-α levels than controls (all *p* < 0.01), and severe COVID-19 pneumonia patients had higher IL-1β and IL-6 levels than moderate cases (all *p* < 0.01)	IL-1 β = 8 (9–57), IL-6 = 32 (13–146), TNF-α = 5.7 (3.5–18)	IL-1 β = 2.3 (0.25–4.15), IL-6= 8 (2–14.7), TNF-α = 1.8 (0.4–2.6)
4	Hamed Valizadeh, 2020, Iran	International Immunopharmacology	Placebo-controlled clinical trial	Covid + ve = 40 (severe, 19–69 yrs), HC = 40 (22–65 yrs)	IL-1β, IL-6, and TNF-α were increased significantly in COVID-19 patients compared with healthy control group (*p* < 0.05)	NR in cases versus controls	NR in cases versus controls
5	Saeid Taghiloo, 2020, Iran	Iranian Journal of Immunology	Cross sectional observational study	Covid +ve = 61 (62 (50–72 yrs), HC = 31, (60.2 yrs)	IL-1β, TNF-α, and IL-6 (all *p* < 0.0001) were higher in cases than controls. Mild and severe. IL1β and TNF-α (*p* < 0.0001), along with IL-6 (*p* = 0.0001) were higher in severe cases than mild cases	NR in cases versus controls	NR in cases versus controls
6	Laura Bergantini, 2022, Italy	Cytokine	Prospective study	Covid + ve = 64 (Mild moderate and severe, 59–67 yrs), HC = 27 (36–78 yrs)	IL-6 was higher in severe cases than HC (*p* < 0.001), no difference between cases and HC in IL-β and TNF-α levels. IL-1β was higher in severe than in mild/ moderate cases (*p* = 0.048; *p* = 0.042) and IL-6 was higher in severe than in mild/moderate cases (*p* < 0.05, *p* < 0.01)	AUC = IL-6 = AUC = 0.70, 95 %CI: 0.57–0.85p = 0.007 (pg/mL)	NR in controls
7	Bandar Alosaimi, 2021, Saudi Arabia	Frontiers in immunology	Observational study	Covid + ve = 53 (Mild and critical, 55 ± 18 yrs), HC = 18, (16–92 yrs)	IL-1β, TNF-α, and IL-6 levels were higher in severe cases than controls (*p* < 0.001), and TNF-α was higher in mild cases than controls (*p* < 0.05)	NR in cases versus controls	NR in cases versus controls
8	Jia Guo, 2022, USA	Jounal of clinical endocrinology and metabolism	case-control (died-survived) study	Covid + ve = 205 (65–72 yrs), HC = 333 (60–68 yrs)	IL-6 was higher in cases than controls (*p* < 0.05) and significantly associated with mortality, whereas no difference in IL-1β and TNF-α levels was seen between cases and controls	NR in cases versus controls	NR in cases versus controls
9	Anbalagan Anantharaj, 2022, India	Journal of Clinical Virology	Observational study	Covid +ve = 16 (26–45 yrs, HC = 10, (26–45 yrs)	No difference in IL-1β and IL-6 between groups, and TNF-α was slightly increased in cases and this was insignificant	NR in cases versus controls	NR in cases versus controls
10	Nihayet Bayraktar, 2021, Turkey	Journal of Medical Virology	Observational study	Covid + ve = 31 (53.72 ± 17.02 yrs), HC = 43, (50–53 yrs)	Levels of all cytokines were higher in the cases than control group	IL-1β= 140.37 ± 64.32, IL-6 = 249.02 ± 62.84, TNF-α = 151.59 ± 56.50	IL-1β = 23.98 ± 11.64, IL-6 = 51.77 ± 21.24, TNF-α = 52.74 ± 20.43
11	Zhen-Zhen Zhang, 2021, China	Pediatric pulmonology	Observational study	Covid +ve = 20 (14.50–17.00 yrs, (mild, moderate, severe), HC = 20, (~14.5 yrs)	IL-1β and TNF-α were decreased more in cases than controls (*p* < 0.05), whereas no difference was found in IL-6	NR in cases versus controls	NR in cases versus controls
12	Mathilda Mandel, 2020, Israel	Cytokine	Prospective, non-randomized study	Covid +ve = 71 (mean 62 yrs), HC = 20, (mean 48.9 yrs)	IL-1β (*p* = 0.03), IL-6, and TNF-α were higher in case than controls, IL-6 and TNF-α were significantly higher in patients that did not survive	IL-1β = 0.67 ± 1.38, 0.31; IL-6 = 117.24 ± 229.48, 39.65; TNF-α = 22.88 ± 12.15, 19.09.	IL1β (0.10 ± 0.15, 0.03); IL6 (1.80+ ± 0.88, 1.61); TNF-α (9.92 ± 2.04; 9.65).
13	Sophie Stukas, 2020, Canada	Critical Care Explorations	Multicenter prospective observational study	Covid + ve = 26, (70 yrs) HC = 22 (65 yrs)	IL-6 and TNF-α (*p* = 0.03) were increased more in cases than controls, however, IL-β was not significantly different among groups	IL-1β = 0.19 (0.16–0.59); IL-6 = 79.9 (27.7–200); TNF-α = 10.2 (6.05–16.9)	IL-1β = 0.23 (0.082–0.40); IL-6 = 65.0 (25.2–154); TNF-α= 6.16 (4.09–10.0)
14	Laura Bergamaschi, 2021, UK	Immunity	Single center cohort study	Covid + ve = 246 (18–60 yrs), HC = 45 (65 yrs)	All cytokines were significantly elevated in cases group than control groups (*p* < 0.0005)	NR in cases versus controls	NR in cases versus controls
15	Arulkumaran, Nishkantha, 2021, India	Critical care explorations	Observational study	Covid + ve = 86 (Mild = 44, Severe = 42, 48–73 yrs), HC = 7 (28–49 yrs)	IL-1β and TNF-α were higher in controls than cases, and IL-6 was higher in cases than controls	NR in cases versus controls	NR in cases versus controls

Table legends: Covid + ve = confirmed COVID-19 patients with RT-PCR.

**Table 3:** Data showing association between periodontitis and COVID-19 and if periodontitis worsens COVID-19 severity.

S No	Author, Year, Country	Journal, Study Type	COVID + ve, COVID-ve, Periodontitis, No Periodontitis Subjects (N)	Association/ No Association	Periodontitis Worsens COVID-19 Disease Outcome
1	Shipra Gupta, 2022, India	Clinical oral investigations, cross-sectional analytical study	82, NR, 65, 27	Association	Yes
2	Pradeep S. Anand, 2021, India	Journal of Periodontology, case-control study	79, 71, 79, Nil	Association	NR
3	Yi Wang, 2021, China	Journal of Translational Medicine, Mendelian randomization study	1299010; NR, 975; NR	Association	Yes
4	Supriya Mishra, 2022, India	Dentistry Journal, cross-sectional study	294, NR, 149, 66	Association	Yes
5	Nora Alnomay, 2022, Saudi Arabia	Saudi Dental Journal, retrospective cohort study	188, NR, 99, 89	Association	Yes
7	Avineet Kaur, 2022, India	Journal of Family Medicine and Primary Care, Comparative study	116, NR, 81% (covid + ve in hospital) 46.2% (covid- + ve home quarantine); NR	Association	Yes
8	Panagiotis Gardelis, 2022, Switzerland	Clinical and Experimental Dental Research, Pilot study	30, NR, 30, NR	Association	Yes
9	Boy M. Bachtiar, 2022, Indonesia	Saudi dental journal, crosssectional study	23, 6, 6 NR	Unclear association	NR
10	Camila Alves Costa, 2022, Brazil	Journal of Periodontology, prospective study	128, NR, 46, 8	Association	Yes
11	H. Larvin,2021, UK	Journal of Dental Research, retrospective longitudinal study	14466, NR, 6631, 35154	Association	Yes
12	S. Wadhwa, 2022, USA	Saudi Dental Journal, retrospective study	387, 387, Unclear, NR	Association	Yes
13	Harriet Larvin, 2020, UK	Frontiers in Medicine, retrospective study	1616, 11637, 2100, 11153	Association	Yes
14	Israel Guardado-Luevanos, 2022, Mexico	International Journal of Environmental Research and Public Health, case-control study	117, 117, 42, 56	Association	Yes

Table legends: Covid + ve = confirmed COVID-19 patients with RT-PCR.

**Table 4:** Scope for further research and unanswered questions.

Scope for Further Research and Unanswered Questions
**Chemical profile** -Report the cytokine range in population of specific age groups -Familial H/o inflammatory comorbidities that influence cytokine level in the same patients with COVID-19 infection/periodontitis -If the COVID-19 infection altered the GCF/gingival cytokine concentration -Cytokine serum level threshold beyond which the patient dies. **Disease outcome** -Respiratory symptoms corresponding to the serum cytokine level -PD/CAL/defect size corresponding to the serum cytokine level -Patterns in bone loss corresponding to the serum cytokine levels -Influence of serum cytokine levels on the disease prognosis **Disease management** -Occurrence of coronavirus in intrabony defects/gingival tissues -Occurrence of periodontopathic bacteria in the lung lesions of COVID-19-infected subjects, and if it is increased with periodontitis severity -If COVID-19/periodontitis increases the susceptibility to other inflammatory conditions -If COVID-19 infection alters the periodontal treatment outcome/tissue healing/dental implant stability -Serum cytokine comparison among COVID-19/periodontitis/periodontitis + COVID-19 to find an association. -If systemic anti-cytokine therapy could decrease disease severity

## Data Availability

Data sharing not applicable. No new data were created or analyzed in this study. Data sharing is not applicable to this article.

## Supplementary Materials

The following supporting information can be downloaded at: https://www.mdpi.com/article/10.3390/biology12040550/s1, Supplementary Table S1: Search keywords, Supplementary Table S2: Excluded studies in periodontitis.
