# Supplementary material for: Is There a Similarity in Serum Cytokine Profile between Patients with Periodontitis or 2019-Novel Coronavirus Infection?—A Scoping Review
Source: Biology (Basel). 2023 Apr 4;12(4):550. doi: 10.3390/biology12040550 (PMC10135784; doi:10.3390/biology12040550)
Supplement: Supplementary file 1 [file biology-12-00550-s001.zip › biology-2284867-supplementary.pdf]

Supplementary table S1 : Search keywords

Covid-19 infection AND IL-1beta AND IL-6 AND TNF-alpha	((((severe acute respiratory syndrome OR SARS OR SARS-CoV OR SARS CoV OR novel coronavirus OR nCoV OR 2019-nCoV OR COVID OR SARS-CoV-2 OR COVID-19 OR Wuhan) OR severe acute respiratory syndrome coronavirus 2/) AND ("tumor necrosis factor inhibitor"[All Fields] OR "TNF inhibitor"[All Fields] OR "tumor necrosis factor inhibitor"[All Fields] OR "tumor necrosis factor-alpha inhibitor"[All Fields] OR "TNF-alpha inhibitor"[All Fields] OR "TNF" [All Fields] OR "TNF-α" [All Fields])) AND (IL-6 OR cytokine OR interleukin OR macrophage activation syndrome OR hemophagocytic lymphohistiocytosis OR HLH) OR exp Cytokines/ OR exp cytokine/ OR exp Interleukins/ OR exp Interleukin-6/ OR exp interleukin 6/ OR exp Receptors, Interleukin-6/ OR exp Macrophage Activation Syndrome/ OR exp Lymphohistiocytosis, Hemophagocytic/ OR exp hemophagocytic syndrome/)) AND ((("interleukin 1"[MeSH Terms] OR "interleukin 1"[All Fields] OR "il 1"[All Fields]) OR ("IL-1B"[All Fields] OR ("interleukin 1beta"[MeSH Terms] OR "interleukin 1beta"[All Fields] OR "il 1 beta"[All Fields]) OR ("interleukin 1beta"[MeSH Terms] OR "interleukin 1beta"[All Fields] OR ("interleukin"[All Fields] OR "1beta"[All Fields]) OR "interleukin 1beta"[All Fields])) OR "IL1B"[All Fields])
Periodontitis AND IL-1beta AND	((((((((aggressive periodontitis) OR chronic periodontitis) OR periapical periodontitis) OR gingivitis) OR periodontitis) OR periodontal disease)) AND ((((Tumor necrosis factor) OR TNF-

IL-6 AND TNF-alpha	alpha) OR Tumor necrosis factor - alpha) OR TNF-α)) AND ((((IL-6) OR Interleukin-6) OR Interleukin-6 receptor) OR IL-6R)) AND (((((((Interleukin-1) OR IL-1) OR Interleukin-1B) OR IL-1B) OR IL-1 beta) OR Interleukin-1β) OR IL1B)
Association between periodontitis and covid-19	(Periodontitis) AND (covid-19)

Supplementary table S2 : Excluded studies in periodontitis.

S.No	No: of studies	Reason for exclusion
1	18	Review articles
2	6	Systematic reviews
3	98	In vitro studies
4	21	Cytokine sampling in GCF
5	13	Periimplantitis
6	39	Animal studies
7	1	Report and guidelines
8	15	Cytokine sampling in saliva
9	6	Cytokine sampling in periapical tissues / fluids
10	10	Cytokine sampling in gingival tissue
11	8	Article in languages other than English

12	3	Cytokine sampling in subgingival plaque
13	16	In vitro analysis using human samples
14	1	Study protocol registration
15	11	No controls included in study design
16	36	Periodontitis along with other systemic conditions
18	1	Combined orthodontic-periodontic treatment for patients with periodontitis
19	3	Pregnant women
